# Self-Management Strategies in Recovery From Mood and Anxiety Disorders

**DOI:** 10.1177/2333393615606092

**Published:** 2015-09-21

**Authors:** Benjamin Villaggi, Hélène Provencher, Simon Coulombe, Sophie Meunier, Stephanie Radziszewski, Catherine Hudon, Pasquale Roberge, Martin D. Provencher, Janie Houle

**Affiliations:** 1 Université du Québec à Montréal, Montréal, Québec, Canada; 2 Université Laval, Québec City, Québec, Canada; 3 Université de Sherbrooke, Sherbrooke, Québec, Canada

**Keywords:** recovery, self-management, depression, anxiety, bipolar disorder, mental health and illness

## Abstract

Mood and anxiety disorders are the most prevalent mental disorders. People with such disorders implement self-management strategies to reduce or prevent their symptoms and to optimize their health and well-being. Even though self-management strategies are known to be essential to recovery, few researchers have examined them. The aim of this study is to explore strategies used by people recovering from depressive, anxiety, and bipolar disorders by asking 50 of them to describe their own strategies. Strategies were classified according to dimensions of recovery: social, existential, functional, physical, and clinical. Within these themes, 60 distinct strategies were found to be used synergistically to promote personal recovery as well as symptom reduction. Findings highlight the diversity of strategies used by people, whether they have depressive, anxiety, or bipolar disorders. This study underscores the importance of supporting self-management in a way that respects individual experience.

## Introduction

Mood and anxiety disorders are the most common mental disorders (Patten et al., 2006; Somers, Goldner, Waraich, & Hsu, 2006). The lifelong prevalence of anxiety disorders is 16.6% (Somers et al., 2006), compared with 12.2% for major depressive disorder (Patten et al., 2006) and 2.2% for bipolar disorder (Schaffer, Cairney, Cheung, Veldhuizen, & Levitt, 2006). These disorders are not only frequent and debilitating (Andrews, 2008; Mathers, Fat, & Boerma, 2008; Revicki et al., 2012), but they are also often concurrent (McElroy et al., 2001; Sanderson, DiNardo, Rapee, & Barlow, 1990).

People living with one of these disorders must learn to live with their illnesses from day to day, particularly as bipolar disorder is a chronic illness, and anxiety and depressive disorders have a very high recurrence rate (Hardeveld, Spijker, De Graaf, Nolen, & Beekman, 2010). To deal with their illness, people with a mental disorder may implement self-management strategies, that is, behaviors intended to control their symptoms, prevent relapses, and optimize their health (Barlow, Wright, Sheasby, Turner, & Hainsworth, 2002). Several studies show that the use of such strategies plays a key role in mental health recovery (Deegan, 2005; Mansell, Powell, Pedley, Thomas, & Jones, 2010; Murray et al., 2011; Russell & Browne, 2005; Sterling, Silke, Tucker, Fricks, & Druss, 2010; Todd, Jones, & Lobban, 2012; Veseth, Binder, Borg, & Davidson, 2012; Young & Ensing, 1999). Individuals with a mental illness are willing and able to actively participate in their recovery, including those who are most affected (Pollack, 1996; Russell & Browne, 2005; Suto, Murray, Hale, Amari, & Michalak, 2010).

Even though self-management strategies are known to be essential to recovery, only a few researchers have examined strategies for fostering the recovery of people with a depressive disorder (Eller et al., 2005; Grieken, Kirkenier, Koeter, Nabitz, & Schene, 2013; Morgan & Jorm, 2009) or a bipolar disorder (Mansell et al., 2010; Michalak et al., 2011; Miklowitz, 2008; Murray et al., 2011; Russell & Browne, 2005), and, to our knowledge, no one has documented the strategies used specifically by individuals with an anxiety disorder. Furthermore, despite the high comorbidity rate of these mental disorders, there has been no investigation of the commonalities among their self-management strategies, even if some authors (e.g., Harvey, Watkins, Mansell, & Shafran, 2004) suggest that such strategies can foster recovery from more than one mental disorder.

We can distinguish two types of recovery in mental health: clinical recovery and personal recovery (Slade, 2009). Clinical recovery can be defined as a reduction in psychiatric symptoms below a clinical threshold (Boschen, Neumann, & Waters, 2009; Dowrick et al., 2011; Ekers, Richards, & Gilbody, 2008; Haro et al., 2011; Mueller et al., 1999). This is the type of recovery that is most often studied in medicine and psychology. In contrast, personal recovery may be defined as “a deeply personal, unique process of changing one’s attitudes, values, feelings, goals, skills and/or roles . . . a way of living a satisfying, hopeful and contributing life even with the limitations caused by illness” (Anthony, 1993, p. 527). This conceptualization of recovery is closer to the views of people with mental illness (Deegan, 1988; Mead & Copeland, 2000; Piat et al., 2009; Ridgway, 2001) and represents the main objective of mental health policy in many countries (Department of Health, 2009, 2011; Mental Health Commission of Canada, 2012; New Freedom Commission on Mental Health, 2003).

To our knowledge, Whitley and Drake’s (2010) dimensional model is the only one that integrates clinical and personal aspects of recovery. It defines recovery on the basis of five inter-related dimensions: (a) clinical—reduction in symptoms; (b) existential—better sense of hope, empowerment, and spiritual well-being; (c) functional—recovering meaningful role; (d) physical—promoting physical health; and (e) social—consolidating relationships with others and feeling that one is part of society. Given its comprehensiveness, Whitley and Drake’s framework provides a useful template for classifying the self-management strategies used by persons with depressive, bipolar, and/or anxiety disorders to recover from their illnesses. Discerning self-management strategies, based on their impacts on the various dimensions of recovery, appear to be not only possible from a theoretical point of view but also essential for practical purposes. By knowing the impacts of the self-management strategies they use to recover, persons with a mental disorder (or their health professional) could identify on which recovery dimensions they are working as well as those they have yet to work on. Furthermore, if Whitley and Drake have delineated the key dimensions of recovery, there is still a lack of knowledge on what people may do concretely to foster these dimensions.

## Objectives

To fill gaps left by prior research, the goals of this study were (a) to explore the variety of self-management strategies used by people to recover from a depressive, bipolar, or anxiety disorder and (b) to describe the strategies fostering recovery in each of the key recovery dimensions.

## Method

### Participants

The sample (*N* = 50) was recruited with assistance from community organizations, health and social services centers, and hospitals. For inclusion in the study, patients had (a) to be 18 years or older; (b) to have lived through or currently be experiencing a depressive, anxiety, or bipolar disorder; (c) to be in recovery or recovered; and (d) to live within a 60 km radius of Montréal, Canada.

As recovery is considered an idiosyncratic process (Chapman, 2002; Russell & Browne, 2005; Young & Ensing, 1999) as well as a continuous process (Anthony, 2003; Deegan, 1996; New Freedom Commission on Mental Health, 2003; Onken, Craig, Ridgway, Ralph, & Cook, 2007), it was left to each participant to decide whether he or she had recovered or was in recovery, and no limit was set on the time between the appearance of the illness and the interview. Potential participants were administered the Patient Health Questionnaire–9 (PHQ-9; Kroenke & Spitzer, 2002), Generalized Anxiety Disorder–7 (GAD-7; Spitzer, Kroenke, Williams, & Löwe, 2006), and the Altman Self-Rating Mania Scale (Altman, Hedeker, Peterson, & Davis, 1997) in a preselection interview to measure symptoms of depression, anxiety, and mania, respectively. Patients with severe scores of depression (PHQ-9 score > 20), anxiety (GAD-7 score > 15), or mania (Altman Self-Rating Mania Scale score > 6) were excluded from the study and directed to appropriate resources for assistance.

The desired composition of the sample targeted comparable proportions of men and women; participants with depressive, anxiety, and bipolar disorders; participants of high and low economic status; and participants living in urban and rural environments. It was important to ensure variety in the sample based on these variables, as gender (Schön, 2010), income (Virtanen et al., 2011), and living environment (Degenhardt, Gatz, Jacob, & Tohen, 2012) may influence self-management strategies and recovery. However, despite a sustained recruiting effort, it was not possible to obtain comparable proportions of participants for the variables of environment and socio-economic status.

The final sample consisted of 24 men and 26 women. The participants averaged 47.3 years of age (*SD* = 10.5 years), and half of them were 46 to 55 years of age. A large majority of them (88.0%) were living with or had experienced more than one mental disorder (depression and anxiety, or anxiety and bipolar disorder). Despite this high level of comorbidity, when we considered only the dominant disorder of each participant, most were mainly living with or had experienced a depressive disorder (42%) or a bipolar disorder (40%). Most of the participants came from urban settings (80%) with an income level above the poverty line (78%).

### Data Collection

An individual interview was conducted with each participant by one of two co-investigators. The interviews took place in the participant’s home or on university premises. All the participants signed a consent form that was approved by the accredited ethics committees. Then the interviewer administered the Mini International Neuropsychiatric Interview (Lecrubier et al., 1997) to identify each participant’s dominant mental illness, the length of his or her illness, and any relapses. Finally, the interviewer asked semi-structured questions inviting participants to describe their self-management strategies. The interviews lasted between 45 and 60 minutes. Each interview was recorded on a digital audiotape and transcribed in its entirety.

The semi-structured questions were developed based on the Critical Incident Technique (Flanagan, 1954). As proposed initially by Flanagan (1954), the technique focused on actual behaviors that people report using to achieve an objective specified by the researcher. This technique has been applied to identify the self-management strategies used by persons living with HIV (Nicholas et al., 2002). Drawing on how the technique was used in Jutras and Morin (2003), participants were asked to talk about (a) the general context of the situation, (b) the action taken or the thoughts that had helped them feel better, (c) reactions from their families and friends as well as professionals/caregivers, and (d) impacts on their thoughts and behaviors. Each participant was asked to describe several critical incidents to elicit the maximum number of self-management strategies, so that exhaustiveness is maximized as required by the technique (Kemppainen, 2000). Although we concentrated our analysis on the actions or thoughts collected with the second question, answers to the other questions were used as criteria for relevance (Woolsey, 1986). The interview guide was successfully pretested with two persons who satisfied the inclusion criteria.

### Data Analysis

Data were transcribed verbatim and, as data collection continued, two coders used NVivo v.10 software to analyze the data. For the present study, a thematic analysis was chosen because the goal was to reveal and organize the various strategies evoked in participants’ answers, without quantifying their exact occurrence as in content analysis (Braun & Clarke, 2006; Vaismoradi, Turunen, & Bondas, 2013). The main steps in the procedure proposed by Braun and Clarke (2006) were followed: getting familiar with the data, generating codes and assigning them to segments of data, combining codes into overarching themes (and sub-themes), reviewing and naming themes, and writing up the results. In addition, an orientation similar to Sandelowski’s (2010) descriptive approach was applied when the data were coded: the level of analysis was maintained as close as possible to participants’ answers, rather than performing deep transformation and interpretation during the analysis. We wanted the strategies reported in this article to stay *participants’ strategies*, rather than becoming a transformed account of their experience.

With respect to the development of codes and themes, in line with Fereday and Muir-Cochrane (2006), we opted for a hybrid orientation involving an inductive analysis followed by the organization of codes around theoretical dimensions from Whitley and Drake’s (2010) model. First, the two coders inductively developed codes for characterizing the self-management strategies emerging from the data. The initial codes were not fixed; rather, they were subject to change as the analysis progressed. In the process, we noticed that the emerging codes could easily be combined under overarching themes that overlapped with dimensions of Whitley and Drake’s framework. As a result, a new coding table (Crabtree & Miller, 1992) was obtained based on this existing theoretical framework. Each of the recovery dimensions corresponded to one theme encompassing several sub-themes describing more specifically the nature of the included self-management strategies.

To compare the self-management strategies used by people with different disorders, we coded each participant’s dominant illness. We then verified which themes were reported by participants from each diagnostic group (depressive, anxiety, and bipolar) and whether there were important differences between these three groups. Chi-square and Fisher tests were performed to verify whether the differences were significant.

### Validity

The research team took various measures to establish the validity of the study. First, to stay close to the participants’ experiences, the interviews were not organized specifically around the dimensions of Whitley and Drake’s (2010) model. Rather, participants were asked to freely describe their self-management strategies and discuss their impact without any knowledge of the study’s underlying theory.

Then the data triangulation strategy was used, meaning that we took into account the points of view of different actors on the development of the coding table and data analysis (Guion, Diehl, & McDonald, 2011). To begin with, the first six interviews were cross-coded by the two coders. For consistency, they also cross-coded two other interviews: one halfway through the coding process and another at the end of the process. An inter-rater agreement of approximately 75% was obtained. In addition to these inter-rater agreements, the two coders met on a regular basis throughout the data analysis period to evaluate the relevance of codes as they were created or modified. Last, upon completing each group of approximately 15 analyzed transcripts, they shared the results of their analysis with the other members of the research team to obtain their points of view on the coding table.

Team members had to reach a consensus on each new theme or sub-theme and each change made to the codes. This approach is frequently used in qualitative research and reduces the bias associated with data analysis (Edwards, Dattilio, & Bromley, 2004). A consensus was established so that the themes (or sub-themes) could be considered mutually exclusive, while maximizing their exhaustiveness.

Finally, new participants were interviewed until a point of data saturation was reached. In other words, data collection stopped when no more themes or sub-themes had to be added when analyzing the data from a new interview. This was important, as we wanted to explore the full diversity of self-management strategies rather than collect data only on the most obvious or frequent ones. Furthermore, it enhanced the trustworthiness of the data (Elo et al., 2014).

## Results

Table 1 provides a detailed listing of the 60 self-management strategies that emerged from the data and that, according to our participants, foster recovery. The strategies mentioned by participants are presented according to which dimensions of Whitley and Drake’s (2010) framework they are fostering.

**Table 1.** Self-Management Strategies Identified in Participants’ Critical Incidents.

Recovery Self-Management Strategies
1. Social
1.1. Surrounding myself with people who make me feel better
Getting support from friends, family, and people with a similar illness
Engaging in activities with others
Choosing the people with whom you can discuss problems
Avoiding negative people or unhealthy relationships
1.2. Taking care of others
Being easy on family and friends
Reassuring family and friends
Serving as a role model for friends and family
Recognizing the support received
Serving others
2. Existential
2.1. Having a positive outlook
Taking inspiration from someone who has recovered
Using downward social comparison
Taking stock of your progress
Remembering times of wellness
Seeing the illness as an opportunity to make some changes in your life
Appreciating positive aspects of your life
Reading or posting inspiring thoughts/images
Having spiritual beliefs
Using humor
Setting aside negative thoughts
2.2. Developing a balanced sense of self
Recognizing and valuing your strengths/achievements
Accepting your limitations/weak points
Accepting the illness
Distinguishing the illness from your personality
Seeing mental illness as equivalent to a physical illness
2.3. Finding meaning
Having realistic expectations about recovery
Finding a project, a goal, a dream
2.4. Empowering oneself
Realizing the efforts required to recover
Finding the motivation needed to recover
Being more assertive about your needs and expectations
3. Functional
3.1. Creating a routine
Following a schedule
Performing daily personal care tasks
3.2. Taking action
Engaging in pleasant activities
Engaging in activities in which you can feel competent
Setting yourself small realistic objectives
Adopting a significant role in society
Respecting your own rhythm as you take action
4. Physical
4.1. Maintaining a healthy lifestyle
Engaging in sport activities
Adopting good sleep patterns
Eating well
4.1. Maintaining a healthy lifestyle (cont.)
Reducing your consumption of stimulants
Reducing your consumption of drugs and alcohol
Stopping smoking
4.2. Managing one’s energy levels
Avoiding stimulating or stressful situations
Engaging in relaxation/breathing exercises
Reducing hours of work
5. Clinical
5.1. Seeking formal professional help
Receiving help from a health professional
Going to the hospital
Receiving help from a mental health organization
Receiving an alternative treatment
Taking your medication
5.2. Developing a better understanding of your illness
Learning about available resources
Attending conferences/workshops
Finding information on mental illness
Investigating the causes of your illness
5.3. Managing daily symptoms
Analyzing and changing your thoughts/emotions/behaviors
Confronting your fears
Gaining some perspective on situations
Looking for solutions to a problematic situation
5.4. Preventing relapse
Remaining vigilant to signs of a relapse/monitoring your moods
Continuing to implement strategies

### Strategies Fostering Social Recovery

Most of the participants mentioned strategies aimed at breaking their isolation and maintaining or developing satisfying social relationships. To this end, many sought support from family, friends, and people with a similar illness. This allowed them not only to receive support and develop new friendships but also to break their isolation. In this respect, one participant said, “It’s so important to know that you aren’t alone dealing with this kind of thing” and “The presence of others with the same condition . . . allows us to feel less isolated.” Some participants also said that they appreciated the comfort provided by the presence of an animal. One participant mentioned how the affection he received from his dog “made all the difference.”

Several participants also became involved in social activities (e.g., meals, sports) to make new contacts or spend time with family and friends. For example, one participant said that playing badminton helped him recover, as it gave him opportunities to socialize with friends. Nevertheless, some participants made an effort to choose the people they socialized with, to find the kind of support they needed. Other participants ended relationships that they considered detrimental to their recovery. As one participant explained,

I’d say that the first thing is to make some changes in terms of the people you’re spending time with. Because if you stay around people who make you suffer, you’ll only continue to suffer. You need to break free of all that; sometimes you’re better off alone than in bad company. I’d also say that when we let go of relationships with people who aren’t good for us, it makes room for people who are better for us.

Furthermore, certain participants implemented strategies focused on taking care of their families or friends. For example, one participant mentioned that he tried to spare his wife and children from his relapses, as he did not want them to “pay a price for my mental health, which isn’t always great.” Other strategies included reassuring family and friends, serving as a role model, and thanking others for the support received. Some participants also said that serving others, whether informally or through volunteer work, helped their recovery. For example, one participant said that discussing her depression at conferences had helped her feeling good, as she believed that by sharing her knowledge, she can help other depressed people recover.

### Strategies Fostering Existential Recovery

Many participants reported using strategies to instill hope for recovery by having a positive outlook. To this end, some participants mentioned that they were inspired by persons who had already been through the recovery process. This included well-known public personalities, family members or friends, or people met at a conference, in a support group, or at a workshop. One participant explained that “if things aren’t going well today, there’s still hope because others have been through difficult times too, and they found that the sun comes up again the next day.”

Some participants became more optimistic after having compared themselves with people whose problems were more severe (downward social comparison). For example, one participant noticed that he felt better after having met people who appeared to be worse off than him. For some participants, it helped to compare themselves with how they felt before, seeing how things had improved. As one participant said, taking stock allowed her to see that “things were going much better” and that she had “come a long way.” Remembering times when one felt well was also mentioned as a good path to becoming more hopeful.

Participants used other strategies to think more positively, such as trying to perceive their illness as an opportunity for personal growth. As one of them said, having a mental illness allowed her to rebuild her life and “not everyone has the luxury of being able to start rebuilding their life from the ground up.” Others tried to appreciate the positive things in their lives, such as family and work relationships, to read encouraging thoughts, and to look at inspiring images. In this respect, one participant said that she had been able to fill two notebooks with quotes and excerpts from her readings. For her, “they are all messages to help me along the way.” Some participants turned to spiritual beliefs, tried to keep their problems in perspective, maintained a sense of humor about themselves, or glossed over negative thoughts to keep hope alive.

To develop a positive self-image, some participants tried to recognize and emphasize their strengths and achievements. To this end, some participants took the time to acknowledge everyday achievements, even small ones. Participants mentioned remembering their talents or recording them on paper to have a more positive perception of themselves. As noted by one participant, this strategy proved to be a big help, because it allowed her to “clearly state my strengths” and understand that “now I feel equipped, I have a toolbox that is all about me.”

Certain participants tried to recognize weaknesses as well as strengths. For example, one participant said that she was trying to stop “banging my head against the wall” when she was unable to work for several hours straight. Other participants tried not to compare themselves with others or apologized for past mistakes. In this regard, one participant said, “I have forgiven myself, I’ve stopped feeling guilty, I’m giving myself a chance.”

Many participants also talked about how it is important to accept their mental illness as a fact of life. As one explained, “First you need to realize that you have a problem. If you don’t, then you can’t solve it.” For many participants, this was a difficult step to take, as it required overcoming one’s shame in having a mental disorder. To help themselves, some participants tried to make a distinction between their mental illness and their personality. As one participant said, “The day that I decided that I was no longer an illness and that I deserved respect, so many things changed; it was a great boost.” Some participants tried to consider their mental illness at the same level as a physical illness, with both biological and genetic bases. As one participant said, “You aren’t any sicker than a diabetic who needs to take insulin everyday. If you take antidepressants, you just take them; it has nothing to do with being crazy.”

To find meaning, some participants also changed their beliefs about recovery. Considering recovery as a long process, or as a process with ups and downs, allowed some participants not to become discouraged in their most difficult moments. As one participant said, recovery “is cyclical; it’s a curve, a wave.” Hence recovery is a mix of good times and more difficult periods.

Having a project, a goal, or a dream also helped some participants find meaning and remain optimistic about the future. As one of them explained,

When I was in Grade 11, I was looking forward to college because I had found a program that I really liked, and that really became my rock. Because I had a goal, I saw myself in the program, I was really interested in it, so even if I wasn’t feeling all that well, I wasn’t necessarily thinking about suicide because I was still able to think a bit about my future.

Highlighting a process of empowerment, some participants mentioned how important it was to appreciate the key role that they played in their own recovery, and the efforts they needed to make to recover. As one participant said, recovery is a process that requires you to “leave the beaten path” and “challenge yourself.” Similarly, another participant stated that “For me, the key thing is that I don’t rely on a group, I don’t rely on a drug, I don’t rely on a therapist. My well-being truly comes from me.” Participants also mentioned the importance of staying motivated and believing in themselves if they were to continue making the efforts needed to recover: “Even if you feel sad or have no more energy, you should still have a desire to get better. Wanting to get better is very important.”

Another strategy used by participants to take charge of their recovery was to be assertive about their needs and expectations. For this purpose, some had to learn to be more expressive. For example, one participant related how, after having told her co-worker that she felt she was treating her disrespectfully, she realized that “In the end, I have a right to say what I think, if something is wrong, even if I’m talking to my doctor or to anyone else in my life. All my life I’ve been afraid to speak up.”

### Strategies Fostering Functional Recovery

Adopting a routine allowed some participants to reduce their symptoms. To this end, participants chose mostly activities that required them to follow a schedule, such as work or studies. For example, one participant said that work “keeps me active” and “forces me to be functional.” Other participants preferred entering activities in their agenda and trying to follow the schedule they had adopted. Performing personal care tasks (e.g., washing, putting on makeup) on a daily basis was also mentioned.

Focused on taking actions, most participants appeared to suffer less from their symptoms when they managed to relax or engage in an enjoyable activity. Many strategies were mentioned in this regard. First, engaging in a pleasant activity appeared to be an effective way to reduce symptoms. This included cultural, artistic, manual, or other activities. For example, one participant said that when she was anxious, she took a “little holiday,” heading out to take some pictures, and this allowed her to lower her anxiety level for a while. Another participant said, “Everyday I give myself at least a half hour . . . I never come straight home (after work); I’ll stop for a coffee and do some reading. I’ll decompress.” Some participants explained that enjoying a recreational activity allowed them to concentrate on something agreeable and to momentarily forget their symptoms. One participant reported that when she was not feeling well, drawing, painting, and “anything that keeps my hands busy” were good distractions.

Some participants mentioned that they had found an activity in which they could use their skills. Work was often mentioned in this context. For example, one participant explained that his work fostered his recovery as he was “extremely well known” in his line of work, and it allowed him to use all his skills.

Regularly setting small objectives is another strategy that allowed our participants to function well. For example, one participant recommended that anyone who wanted to recover should concentrate on “concrete steps, small steps that you can take to pull through.” Assuming significant roles also helped many participants in their recovery. This included serving as a volunteer or focusing on work. One participant related that his activism gave him a “deeply pleasing” and “gratifying” feeling, as he had the impression that “it’s good for everyone, for nature, for me,” and that it was consistent with his most deeply held values. Similarly, for some participants, their role as a parent was the most important thing in their lives.

Some participants stated that it is also important to respect their rhythm as they take action: take their time, trust themselves, and wait for the right moment. One participant chose to wait to “be 100% better” before she returned to work, even if a physician did not have the same opinion. Another learned to pay attention to how she was feeling and adjusted her approach to work accordingly: “If one day I’m not feeling well, I won’t give myself a series of things to do if they can wait. I’ll change my priorities, adjust things to how I’m feeling at that time.”

### Strategies Fostering Physical Recovery

Many of the strategies described by participants specifically concerned physical recovery. In particular, many developed or tried to maintain a healthy lifestyle. Often they spoke of engaging in a sporting activity. One participant reported that for him, physical activity—whether high or low intensity—served as a “natural antidepressant.” Some participants also reduced their symptoms by adopting a good sleep schedule and eating well. As one participant noted, going to bed at a regular time and eating three meals a day reduced the risk of “going from one extreme to another.” Similarly, either reducing or stopping their consumption of stimulants (e.g., coffee), drugs, or alcohol helped some participants manage their symptoms. One participant stated that his consumption of drugs was the cause of his lingering depression. One participant with bipolar disorder found that she needed to stop consuming coffee when she was in a manic episode. Stopping smoking was also mentioned as a strategy.

In addition to general health behaviors, several participants reported strategies focused on managing their energy, such as avoiding stressful situations, performing relaxation exercises (e.g., yoga, Tai Chi, meditation, breathing exercises), taking some time off (e.g., a vacation), or working fewer hours.

### Strategies Fostering Clinical Recovery

In terms of clinical strategies directly aiming at reducing symptoms, most participants noted the importance of seeking formal professional help. This professional assistance came from a physician, a psychologist, a social worker, a peer helper, a facilitator at a mental health workshop, or someone working at a help line. As one participant noted, by seeking “psychological help,” people can “work on the things that are the real sources of their suffering” and thereby reduce symptoms. Some participants mentioned that they sometimes needed to go to a hospital. Besides turning to traditional mental health resources, some participants also relied on alternative resources such as acupuncture, art therapy, lithotherapy, or luminotherapy to help them recover.

Whether or not the participants were favorable to medication, most of them recognized this strategy as effective to reduce symptoms. One participant said that “as soon as you’ve received a drug, you’re able to get some sleep, and that helps alleviate the anxiety.” Another participant observed that the medication eliminated “intense moments” and allowed her to “see clearly without being overwhelmed by a wave of emotion.”

Many participants also sought to better understand their mental disorder and the symptoms they were feeling. Some mentioned that they tried to find resources, attend conferences or workshops, or collect information on mental illness (written material or from health professionals). For example, one participant mentioned that the various documents he had read on mental illness had helped him better recognize his symptoms.

For some participants, thinking about the causes of their mental illness helped them reduce their symptoms. One participant spoke of the moment that she became aware that her illness was due in part to a difficult childhood: “It explained so many inconsistencies in my life. All of a sudden, this new awareness made everything feel easy. Nothing stressed me anymore.”

Various strategies were also used to manage daily symptoms. One of the most often reported was analyzing and changing dysfunctional ways of thinking or behaving. For example, one participant explained that when she analyzed her “little internal conversations,” she started by asking herself whether she was “engaging in black-and-white thinking.” If she was, then she tried to dedramatize the situation and adopt a more nuanced view, to avoid being overcome by negative emotions.

Another strategy often reported by our participants was confronting one’s fears and stepping back from related thoughts. By examining their fears, the participants found that they could gradually gain a different perspective and find solutions to reduce the impact on their emotions and behaviors. As one participant recounted, not confronting what makes you uncomfortable will, over the long term, generate more stress than facing up to them. Not avoiding conflictual situations was also an important strategy for some participants. As one said, it was important to deal with one’s problems in order to no longer suffer the emotional consequences of certain difficult situations. Other strategies used by participants to gain some perspective over difficult situations included drafting a list of pros and cons, taking some time to reflect, not worrying about certain irritants, dedramatizing situations, and working to solve problems.

For preventing relapse, many participants recognized the importance of paying attention to their moods and perceiving the early signs of a relapse. Some participants kept a mood journal, in which they regularly took notes on mood changes. This allowed them to prevent relapses or avoid having their condition deteriorate by implementing an appropriate strategy as soon as they became aware that their symptoms were becoming more intense. For example, one participant explained that when she felt that she was entering a depressive episode, she made an effort to sleep only 7 or 8 hours per night and take a nap during the day, to avoid sleeping 12 hours at a time. Similarly, some participants mentioned the need to stay vigilant throughout the recovery process and to continue to apply one’s self-management strategies.

### Differences Between the Three Diagnostic Groups

Each overarching theme of self-management strategies was mentioned by a large proportion of participants from each diagnostic group. Only two significant differences emerged. Strategies related to “creating a routine” were more mentioned by people with bipolar disorder than by those with anxiety disorders (Phi = +0.41, *p* < .05). Strategies related to “managing daily symptoms” were more mentioned by people with anxiety disorders than by those with depressive disorder (Phi = −0.043, *p* < .05).

### Note on the Overall Pattern of Use of These Strategies

Participants insisted on the importance of identifying their own “recipe for success,” meaning the strategies that work best for them. A participant explained, “If it works for me, that doesn’t mean that it will work for somebody else.” To find the strategies most favorable to their recovery, they had to not be afraid to try new things or, in the words of one participant, you can simply ask the following question: “What do I feel like doing?”

A last finding concerns the synergistic use of the above-mentioned self-management strategies. The results revealed that the participants simultaneously implement strategies tied to the clinical dimension from Whitley and Drake (2010) as well as to the other more personal dimensions of the model. This observation also applies to the use of various strategies within the dimensions. In sum, self-management strategies seem to work synergistically to foster recovery. As one participant explained,

I can say that my medication is very important. But it’s a group of things, a recipe. It’s like a cake that you’re trying to get to rise; it isn’t just one thing that’s key to recovery. It’s truly a group of actions that we take; the drug on its own won’t be enough . . . It really takes a dose of all these things.

## Discussion

This study identifies many, varied self-management strategies used to foster recovery as described by individuals with anxiety, depressive, or bipolar disorders. We identified a total of 60 distinct self-management strategies delineated in several themes and sub-themes. The large number of distinct strategies—given the number of participants—shows the individualized nature of mental health self-management. Although the themes or sub-themes identified here converge with dimensions from the literature, they do not constitute a finished list of strategies that apply or should be prescribed to everyone. The strategies are those experienced as useful by the participants in this specific study, and recovery is a very personal process. Nevertheless, achievement of data saturation suggests that the list is relatively exhaustive.

In line with previous research, self-management strategies fostering social recovery highlight the importance of peer relationships and social activities (Mezzina et al., 2006), moving beyond the sole receiving of support toward reciprocal relationships in which opportunities for giving are also recognized (Tew et al., 2011). A noteworthy finding is the perceived contribution made by pets as sources and recipients of affection, an aspect of support that is garnering progressively more attention (Amiot & Bastian, 2015; Davidson & Stern, 2013).

Concerning strategies related to existential recovery, the person’s belief that recovery is possible is strengthened not only when he or she identifies with role models but also when he or she makes downward comparisons between that person and others. This is consistent with the theory of social comparison (Festinger, 1954), which suggests that downward comparison is one means for feeling better about oneself. Strategies promoting existential recovery also involve both optimizing personal strengths and taking into account one’s limitations. Such balanced self-knowledge has been found to be essential if the person is to be committed to achieving life goals that depend on his or her assets more than his or her weaknesses (Provencher, Gregg, Mead, & Mueser, 2002). Participants also implemented a variety of strategies to foster greater control over the recovery process, such as believing in oneself or setting small objectives. These strategies enhance the person’s desire to take care of himself or herself and improve his or her skills to do so, in line with the conceptualization of empowerment as a motivational process (Conger & Kanungo, 1988).

With respect to strategies fostering functional recovery, one of our most striking findings is the perceived contribution made by concrete activities that allow an individual to play a role in society (e.g., parenting, paid or volunteer work). In other words, the individual is able to maintain or regain a sense of functioning through actions that are meaningful to that person and others. This provides additional empirical support for person-centered care planning, based on functional and meaningful life goals (Tondora, Miller, Slade, & Davidson, 2014).

Regarding strategies fostering physical recovery, the study findings call attention to health behaviors (e.g., physical activity, healthy food, smoking reduction) for managing the symptoms of depressive, anxiety, and bipolar disorders. This is especially important when one considers the comorbidities of physical illness in persons with mental illness (Druss & Walker, 2011) as well as diagnostic overshadowing among this population, neglecting the assessment of physical problems and the provision of physical care (Corker et al., 2013).

Clinical recovery encompasses a large variety of strategies. This includes well-known strategies, such as medication, illness education, cognitive restructuring, and professional support. The participants also used alternative medicines (e.g., meditation, yoga, Tai Chi), which are receiving increased attention in recovery and positive psychology interventions (Johnson et al., 2009; Russinova, Wewiorski, & Cash, 2002).

Taken together, the study’s results represent a significant step forward scientifically for four main reasons. First, compared with prior studies, they offer a broader range of self-management strategies used by persons with depressive or bipolar disorders (Chapman, 2002; Eller et al., 2005; Grieken et al., 2013; Mansell et al., 2010; Miklowitz, 2008; Morgan & Jorm, 2009; Russell & Browne, 2005; Suto et al., 2010; Veseth et al., 2012). Second, the results fill a gap in the literature by exploring the self-management strategies used by persons with anxiety disorders. Third, the study’s results underscore similarities in the self-management strategies reported by persons with a depressive, bipolar, or anxiety disorder. All the broad self-management themes were evoked by a similar proportion of participants from each diagnostic group, with two exceptions. “Creating a routine” was more reported by participants with bipolar disorder, which was not surprising given the importance of routine for preventing recurrence in this disorder (Frank, Gonzalez, & Fagiolini, 2006). Including cognitive behavioral techniques recommended for anxiety (e.g., exposure to fears; Butler, Fennell, & Hackmann, 2008), strategies related to “managing daily symptoms” were more often reported by participants with anxiety disorders. Nonetheless, the overall similarity in strategies used by the three groups suggests that the recovery experience may serve as a rallying point for people with different disorders. Last, the study extended Whitley and Drake’s (2010) framework by providing a comprehensive list of real-life strategies that could promote each dimension.

### Practical Implications

In describing their diverse self-management strategies, the participants demonstrated an ability to control and take charge of their own recovery, and they were aware of the importance of this involvement. In other words, they knew that the outcome would depend on the efforts that they were prepared to make.

Health professionals should be sure to support the empowerment of persons in recovery, as suggested by recent studies (e.g., Deegan, 2005; Todd et al., 2012). This implies the need to regularly inquire about the recovery strategies used by patients (Deegan, 2005) while encouraging efforts to develop new strategies (Veseth et al., 2012). Supporting the person’s ability to act is not incompatible with the use of medical care (Sterling et al., 2010). Most of the participants in this study underscored the important role played by physicians and medication in a successful recovery. Some people may prefer having their professionals make certain decisions, such as medication decisions (Bird et al., 2014). In sum, fostering the empowerment of persons in recovery does not mean letting them just “go at it alone,” but rather allowing them to participate in “who does what” decisions.

Of particular relevance is the fact that symptom self-management strategies (clinical dimension) were not broadly reported more frequently than strategies targeting other dimensions of recovery. Professionals should thus support the self-management of symptoms while also paying attention to other personal issues involved in recovery. In this sense, Whitley and Drake’s (2010) model can be used to structure the support provided by targeting key aspects of personal recovery as well as the symptoms of the mental disorder.

### Future Research

Our research team is highly involved with mental health users and professionals in developing research and practice tools based on the self-management strategies reported by participants in the present study. We developed a research instrument measuring self-management (see Coulombe et al., 2015) intended to be used primarily for research purposes, such as investigating the effectiveness of self-management support programs for which systematic evidence is lacking (Houle, Gascon-Depatie, Bélanger-Dumontier, & Cardinal, 2013). We also are designing an empowerment-based self-management intervention aiming to help people with depressive, anxiety, and bipolar disorders prepare and implement a personalized self-management plan. The efficacy of this new intervention will be assessed in a randomized control trial.

The study has broader implications for future research. It could be interesting to examine the interrelationships between different self-management strategies used along the recovery journey. In this regard, the experiential changes of recovery are sometimes grouped in distinct phases, with movement from one phase to another indicating progress (e.g., see Andresen, Oades, & Caputi, 2011). The participants in this study considered themselves recovered or on a path to recovery and used both clinical self-management strategies and strategies targeting personal dimensions of recovery. One can infer that for our participants, clinical self-management was no longer as important because these strategies had allowed them to encapsulate the illness and move on to other key aspects of their recovery. This raises the possibility that specific categories of strategies predominate during certain recovery phases.

### Limitations

Among the methodological limitations of this study, we note the bias associated with recruiting participants. Only persons who considered themselves recovered or on a path to recovery were interviewed. Even though this sample was appropriate given the study’s objectives, it is not representative of persons starting down a path of recovery. Consequently, the strategies identified in this study must be seen as winning strategies, used by persons who had experienced some success in their recovery process. Also, most participants were living above the poverty line and in urban settings; hence, they may have benefited from an environment favorable to the development and use of certain self-management strategies. Furthermore, given our limited sample size and the small number of participants in recovery from an anxiety disorder, our comparison between diagnostic groups needs to be interpreted with caution.

The use of a predefined theoretical model served to orient the process by which we categorized strategies. In analyzing the transcripts, the researchers may have unconsciously taken steps to validate this model. However, the participants were free to describe the impacts of their strategies without any knowledge of the study’s underlying model. Furthermore, the researchers remained open to the possibility that strategies could be classified outside of this model’s five dimensions. In fact, Whitley and Drake’s (2010) framework is only one source for deriving themes for organizing self-management strategies. Another framework (Leamy, Bird, Le Boutillier, Williams, & Slade, 2011) was considered during the analysis but Whitley and Drake’s model was preferred because of its comprehensiveness.

## Conclusion

Persons living with mood and anxiety disorders used many diverse self-management strategies in their recovery process. Many of these strategies were aimed at personal aspects of recovery (such as existential or social), in contrast with other strategy inventories that are almost exclusively focused on self-management of the clinical symptoms or their impact on the functioning level. This comprehensive view of self-management allowed us to establish similarities among the strategies used by persons with different psychiatric diagnostics—depressive, anxiety, and bipolar disorders—hence fostering the interest in a demedicalized approach centered on the experiences of persons in recovery.
